# Exogenous IL-2 delays memory precursors generation and is essential for enhancing memory cells effector functions

**DOI:** 10.1016/j.isci.2024.109411

**Published:** 2024-03-04

**Authors:** Shaoying Wang, Margaux Prieux, Simon de Bernard, Maxence Dubois, Daphne Laubreton, Sophia Djebali, Manon Zala, Christophe Arpin, Laurent Genestier, Yann Leverrier, Olivier Gandrillon, Fabien Crauste, Wenzheng Jiang, Jacqueline Marvel

**Affiliations:** 1 Centre International de Recherche en Infectiologie, INSERM, U1111, Université Claude Bernard Lyon 1, CNRS, UMR5308, École Normale Supérieure de Lyon, Université de Lyon, Lyon, France; 2 Shanghai Key Laboratory of Regulatory Biology, Institute of Biomedical Sciences and School of Life Sciences, East China Normal University, Shanghai, China; 3 AltraBio, Lyon, France; 4 Inria, Villeurbanne, France; 5 Laboratoire de Biologie et de Modélisation de la Cellule, Université de Lyon, ENS de Lyon, CNRS UMR 5239, INSERM U1210, Lyon, France; 6 Laboratoire MAP5 (UMR CNRS 8145), Université Paris Cité, Paris, France; 7 Faculté de Médecine Lyon-Sud, Université de Lyon, Oullins, France

**Keywords:** Biological sciences, Molecular biology, Immunology, Immune system evolution

## Abstract

To investigate the impact of paracrine IL-2 signals on memory precursor (MP) cell differentiation, we activated CD8 T cell *in vitro* in the presence or absence of exogenous IL-2 (ex-IL-2). We assessed memory differentiation by transferring these cells into virus-infected mice. Both conditions generated CD8 T cells that participate in the ongoing response and gave rise to similar memory cells. Nevertheless, when transferred into a naive host, T cells activated with ex-IL-2 generated a higher frequency of memory cells displaying increased functional memory traits. Single-cell RNA-seq analysis indicated that without ex-IL-2, cells rapidly acquire an MP signature, while in its presence they adopted an effector signature. This was confirmed at the protein level and in a functional assay. Overall, ex-IL-2 delays the transition into MP cells, allowing the acquisition of effector functions that become imprinted in their progeny. These findings may help to optimize the generation of therapeutic T cells.

## Highlights

• Following activation, CD8 T cell initial proliferation is independent of ex-IL-2
• Ex-IL-2 promotes the acquisition of an effector phenotype by activated CD8 T cells
• Ex-IL-2 delays the generation of memory precursor cells
• Ex-IL-2-activated cells give rise to memory cells with enhanced functional traits

## Graphical abstract

**Figure:**
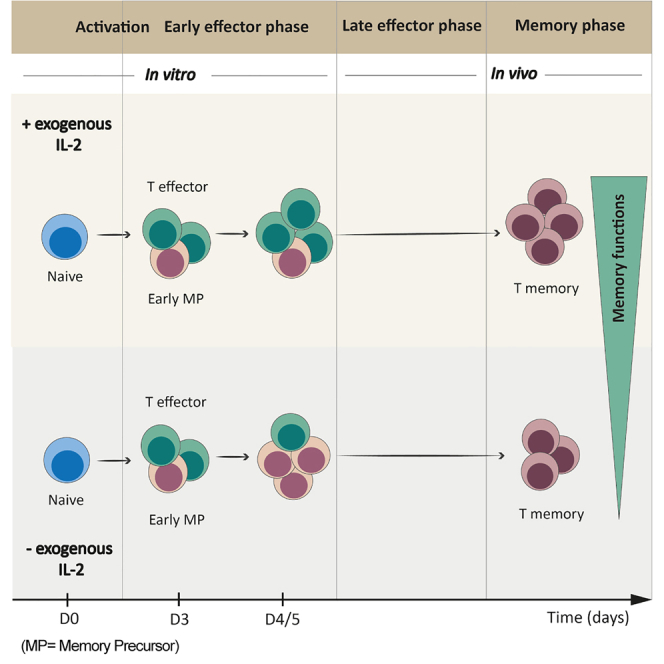

## Introduction

The effectiveness of CD8 T cells responses relies on the generation of effector and memory cell subsets contributing to immune control and long-term protection. Optimal activation of naive CD8 T cells is thus essential and requires 3 signals which are T-cell antigen receptor (TCR) activation, co-stimulation signals, and inflammatory cytokines.^1^ Besides these signals, IL-2 plays a central role in cellular proliferation, survival and also in the differentiation of CD8 T cells.^2^

Its impact on memory differentiation has been extensively studied.^3^^,^^4^^,^^5^^,^^6^^,^^7^^,^^8^^,^^9^ IL-2 is especially essential for the generation of functional memory CD8 T cells since CD25-KO memory cells are non-functional and unable to mount an effective recall response.^7^^,^^9^ However, the role of IL-2 is ambivalent as too much IL-2 signaling seems to counteract the capacity of CD8 T cells to differentiate into memory cells.^2^ Indeed, Kalia et al., using the CD25 expression level as a surrogate for the IL-2 signaling strength perceived by CD8 T cells responding to a viral infection, have shown that CD25^high^ CD8 T cells tend to differentiate into terminal effector cells, while CD25^low^ cells give rise to memory cells.^5^ This indicates that a strong IL-2 stimulation drives CD8 T cells toward terminal effector differentiation. These results are in line with experiments showing that high IL-2 concentrations promote the expression of the effector molecules perforin and granzyme B (GzmB).^7^ Moreover, IL-2 can be produced by both CD4 and CD8 T cells and thus can act on CD8 T cells in a paracrine or autocrine fashion.^2^ The autocrine signal has been shown to be critical for optimal secondary expansion of memory T cells during primary expansion.^3^^,^^8^^,^^10^ This essential role of CD8-derived IL-2 in the generation of memory CD8 T cells has recently been confirmed by tracing the fate of IL-2-producing CD8 T cells *in vivo*. Indeed, over the course of an infection, not all CD8 T cells produce IL-2 and it was found that IL-2 producing-CD8 T cells are more prone to differentiate into memory cells while T cells that do not produce IL-2 instead gain effector cell traits.^4^ Finally, the duration and strength of IL-2 signaling can also direct the effector/memory fate decision. Hence, a prolonged IL-2 signal or a high concentration in IL-2 promotes the effector differentiation whereas a low dose is in favor of a memory phenotype.^5^^,^^7^

The number of responding cells is another parameter that can influence CD8 T cell differentiation. Particularly, CD8 T cells tend to differentiate into central memory cells (TCM) in adoptive transfer experiment when a high number of naive CD8 T cells is transferred.^11^^,^^12^^,^^13^ Similarly, the generation of CD44^+^CD62L^+^ precursors of central memory cells (pTCM) is promoted when naive CD4 T cells are cultured at a high density.^14^

In this context, we reexamined the impact of exogenous IL-2 (ex-IL-2) on the early CD8 T cells activation and the generation of memory precursor (MP) CD8 T cells *in vitro*. We showed that the cellular concentration of responding CD8 T cells determines the dependency on ex-IL-2 for their optimal expansion. We assessed the capacity of these *in vitro* activated CD8 T cells to differentiate into memory cells either directly after adoptive transfer into naive recipients or following *in vivo* re-stimulation into virus-infected mice. We found that ex-IL-2 promotes direct differentiation into memory of a larger fraction of activated CD8 T cells which displayed enhanced memory functional traits when transferred into naive mice, whereas it does not impact their differentiation in time-matched infected mice. We performed single-cell RNA sequencing (scRNA-seq) experiments and found that the transcriptional program of CD8 T cells activated *in vitro* with or without ex-IL-2 was similar on day 3 and only started to diverge from day 4 onwards. Indeed, the majority of CD8 T cells activated in the absence of ex-IL-2 acquired a quiescent-cell gene expression profile and a significant fraction was enriched in a MP signature. In contrast, cells activated with ex-IL-2 maintained a cycling-cell gene expression profile and acquired a gene expression signature associated with effector functions. In line with these results, the supplementation with ex-IL-2 sustained the expression of effector proteins and thus gave rise to cells with greater cytotoxic capacities.

## Results

### Ex-IL-2 has no impact on CD8 T cells proliferation but sustains the expression of CD25 and Bcl-2

**Figure 1:**
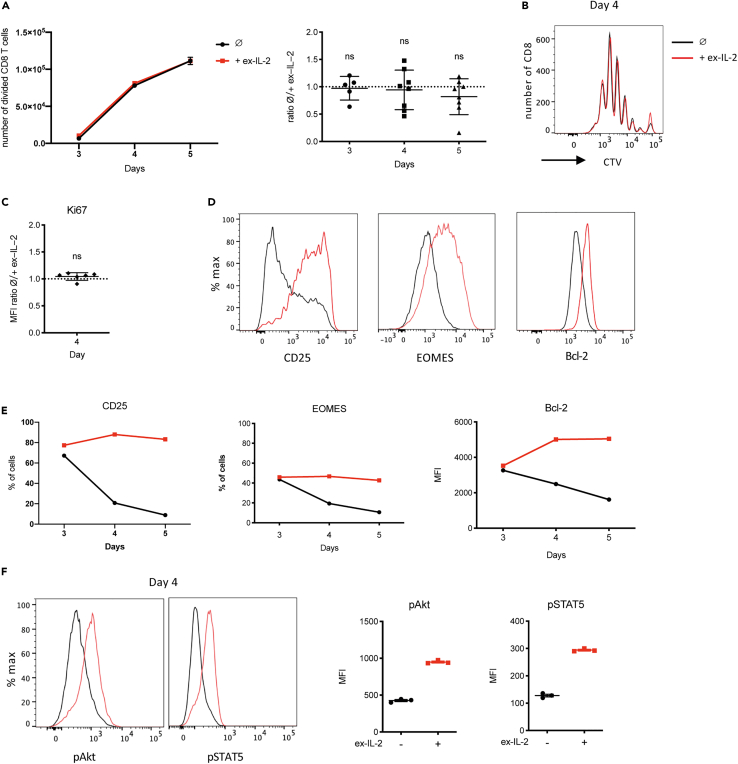
Ex-IL-2 impacts the phenotype but not the proliferation of *in vitro* activated CD8 T cells See also Figure S1. (A–F) 1.5 × 10^5^ magnetically purified naive F5 CD8 T cells labeled with CTV (CellTrace Violet) were cultured with CpG-matured, peptide-loaded cDC at a ratio of cDC:CD8 = 1:10, in the presence or absence of ex-IL-2 (11,5 ng/mL). The strategy to gate divided CD8 T cells is described in Figure S1A. (A) **Left:** The number of divided CD8 T cells (that has undergone at least one division) was determined on days 3, 4 and 5. **Right:** The cell number ratio between cells cultured in the absence or presence of ex-IL-2 was calculated. (B) CD8 proliferation in the presence (red) or absence (black) of ex-IL-2 was analyzed after 4 days by CTV dilution and is represented as overlay histogram. (C) Median fluorescence intensity (MFI) of Ki67 was measured on divided cells after 4 days of activation and the MFI-ratio between cells cultured in the absence or presence of ex-IL-2 was calculated. (D) Expression of CD25, EOMES and Bcl-2 by divided CD8 T cells was analyzed 4 days after activation. Representative histograms of cells cultured in the presence (red) or absence (black) of ex-IL-2 is shown. (E) Kinetics of the percentage of EOMES^+^ and CD25^+^ cells, as well as the level of Bcl-2 expression by divided CD8 T cells. (F) Expression of pAkt and pSTAT5 by divided CD8 T cells were analyzed 4 days after activation. Representative histograms (left panel) and MFI (right panel) of cells cultured in the presence (red) or absence (black) of ex-IL-2 are shown. The mean ± SEM of triplicate cultures from one representative experiment out of at least five independent experiments is shown in panel A (right)-B-D-E, and one out of two in panel F. The mean ± SD of five and six experiments is shown in panel A (Left) and C, respectively. The statistical significance of the difference between the mean value of ratios and the hypothetical value of 1 was determined by the one sample t-test.

To characterize the impact of exogenous IL-2 (ex-IL-2) on CD8 T cell priming, naive F5 CD8 T cells labeled with CTV were activated *in vitro* with NP68-loaded DCs in the presence or absence of ex-IL-2. We measured cell division and characterized the phenotype of CD8 T cells at different time points following activation. Ex-IL-2 had no impact on the number of divided cells recovered 3, 4 and 5 days after activation (Figure 1A left panel) and the ratio between cells numbers in culture with or without ex-IL-2 is maintained over time (Figure 1A right panel). In agreement with these results, the number of divisions and the expression level of the proliferation marker Ki67 by divided CD8 T cells were similar in the presence or absence of ex-IL-2 (Figures 1B and 1C). Furthermore, ex-IL-2 had no impact on the increased CD8 T cell glucose uptake that follows activation (Figure S1B). Increasing the dosage of IL-2 from 11.5 ng/mL to 34.5 ng/mL using two different sources of IL-2 did not influence the number of divided CD8 T cells nor CD25 expression (Figure S1C). These results were not due to the usage of TCR transgenic CD8 T cells since similar results were obtained with non-transgenic polyclonal CD8 T cells from C57BL/6 mice following their activation with anti-CD3 and anti-CD28 coated-beads (Figure S1D). We observed that CD8 T cells produced IL-2 following antigenic stimulation (Figure S1E), suggesting that, in our conditions, intrinsic IL-2 (int-IL-2) might be sufficient to sustain their initial proliferation. From day 4 onwards, ex-IL-2 did, however, impact activated CD8 T cells as it was able to maintain an increased expression of CD25, EOMES and Bcl-2 (Figures 1D, 1E, and S1F) and the phosphorylation of Akt and STAT5 in activated CD8 T cells (Figure 1F). These findings suggest that although ex-IL-2 impacts the expression of EOMES, CD25 and Bcl-2, and maintains the phosphorylation of Akt and STAT5, it does not affect the initial proliferation nor the number of activated CD8 T cells recovered after 4 days of activation.

### The responding CD8 T cell concentration influences the dependency on ex-IL-2

**Figure 2:**
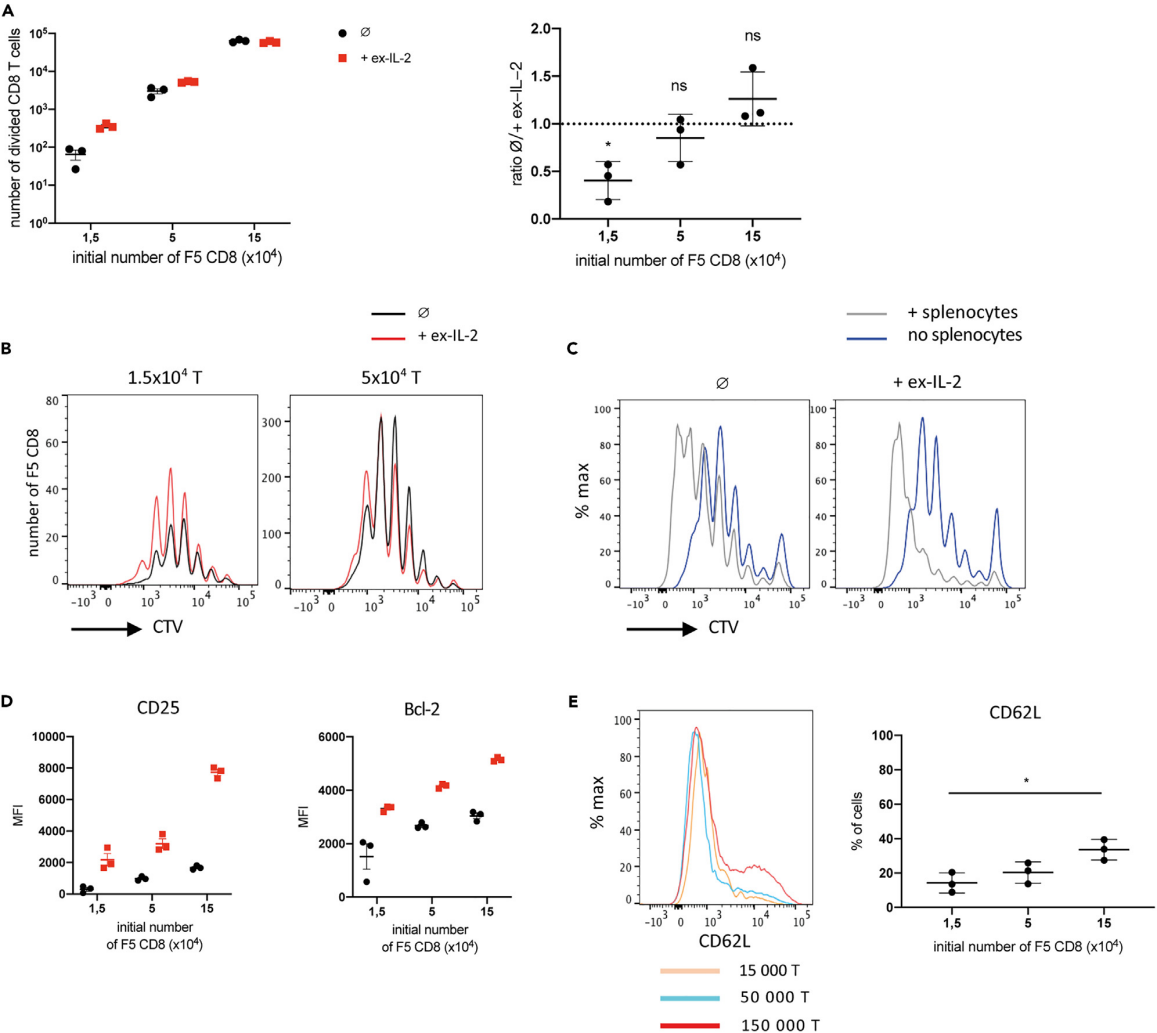
Lower CD8 cellular concentration strongly increases the dependency on ex-IL-2 See also Figure S2. (A, B, D, E) 1.5 × 10^5^, 5 × 10^4^ or 1.5 × 10^4^ purified naive F5 CD8 T cells labeled with CTV were cultured with CpG-matured, peptide-loaded cDC at a ratio of cDC:CD8 = 1:10, in the presence or absence of ex-IL-2 (11,5 ng/mL) for 4 days. (A) **Left:** The number of divided CD8 T cells (that has undergone at least one division) was determined for each cell concentration. **Right:** The cell number ratio between cells cultured in the absence or presence of ex-IL-2 was calculated for each cell concentration. (B) CD8 proliferation in the presence (red) or absence (black) of ex-IL-2 was analyzed after 4 days by CTV dilution and represented as overlay histograms. (C) 5 × 10^4^ purified naive F5 CD8 T cells labeled with CTV were activated as in (A), in the presence or absence of 3 × 10^5^ C57BL/6J splenocytes, and their proliferation was analyzed by CTV dilution 4 days later. (D) Median fluorescence intensity (MFI) of CD25 and Bcl-2 was measured on divided cells for each cell concentration. (E) Expression of CD62L by CD8 T cells activated at different concentrations with ex-IL-2. Representative histograms (Left) and individual percentages of positive cells (Right) are shown. The mean ± SEM of triplicate cultures from one representative experiment out of three independent experiments is presented in panel A (right) and D. The mean ± SD of three independent experiments is shown in panel A (Left) and E. In A (Left), the statistical significance of the difference between the mean value of ratios and the hypothetical value of 1 was determined by the one sample t-test (A). In E, the statistical significance of differences was determined by one-way ANOVA followed by Tukey’s post-hoc test (ns = p > 0.05, ∗ = p ≤ 0.05).

A number of studies showed that the local responding-T-cell density can modulate T cell differentiation.^11^^,^^12^^,^^13^^,^^14^ Therefore, we investigated the impact of ex-IL-2 on the proliferation and differentiation of CD8 T cells activated at different cellular densities. The CD8 T cell concentration at the start of the culture was decreased by 3- and 10-fold, while maintaining a DC:T ratio of 1:10. We observed that decreasing the CD8 T cell density reduced the number of divided CD8 T cells in the presence, as well as in the absence of ex-IL-2, indicating a degree of cellular cooperation independent of IL-2 (Figure 2A). However, ex-IL-2 was able to increase the number of divided CD8 T cells recovered 4 days after activation in a cell density-dependent manner, with low density-cultured cells being especially sensitive to ex-IL-2. To test if the reduced proliferation index was due to a decreased survival of cells when grown at low density, we performed the same experiment adding an excess (3x10^5^) of C57BL/6J splenocytes to the culture. When grown at low density, a similar decrease in the CD8 T cell proliferation index was observed and ex-IL-2 was able to increase the number of divided CD8 T cells recovered (Figure S2B). This suggests that the ex-IL-2 dependency at low cell density was determined by the number of responding cells rather than the number of total cells in the environment. At the lower cell density, the increased number of divided cells was associated with an increased survival of cells having performed higher number of divisions (Figure S2A), which resulted in an increased number of cells in these peaks, but the number of divisions performed was not affected (Figure 2B). However, the presence of non-transgenic splenocytes increased the number of divisions at all cell densities, regardless of the addition of ex-IL-2 (Figure 2C). Interestingly, this was not due to an increase in the production of IL-2 by non-transgenic-spleen cells or CD8 since the IL-2 concentration was lower in the presence of splenocytes (Figure S2C). The reduction of cell density or the addition of non-transgenic splenocytes did not influence the effect of ex-IL-2 on the expression of Bcl-2 or CD25 (Figures 2D, S2D, and S2E). In agreement with what has been observed for CD4 T cells,^14^ the expression of CD62L decreased as cell density decreased (Figure 2E). In conclusion, the activation of CD8 T cells is highly dependent on cellular cooperation and the cellular density of responding cells in the culture influences the dependency on ex-IL-2.

### CD8 T cells activated with or without ex-IL-2 have a similar ability to differentiate into effector and memory cells *in vivo*

**Figure 3:**
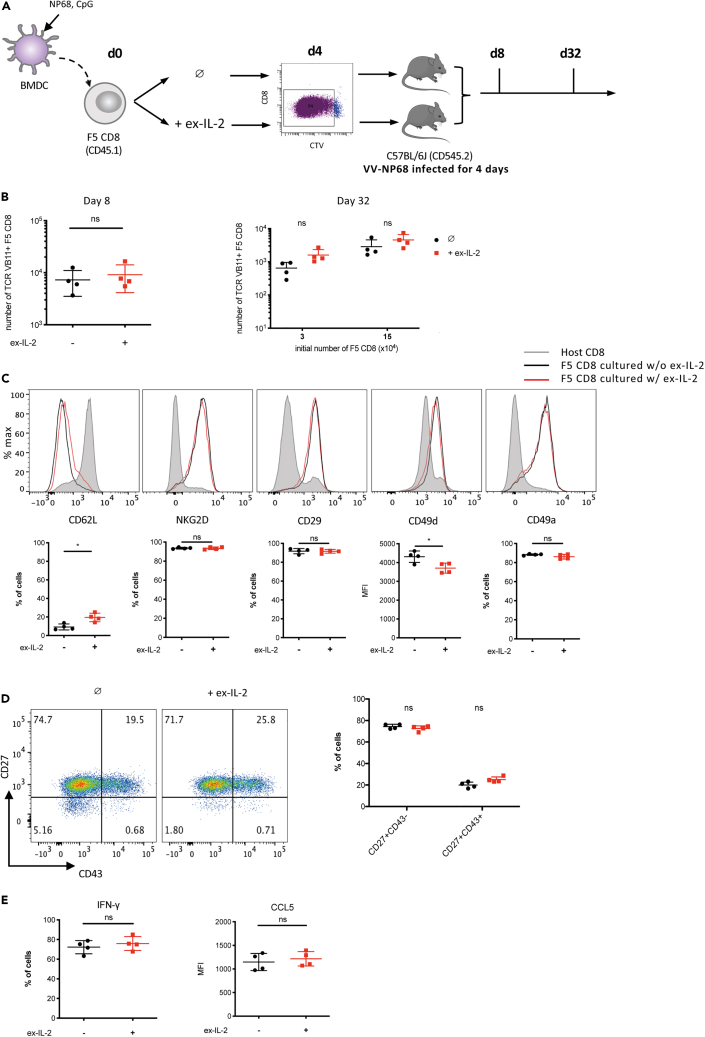
Cells activated with and without ex-IL-2 have a similar potential to participate in an ongoing immune response See also Figure S3. (A–F) CTV-labelled purified naive F5 CD8 T cells, at a concentration of 6 × 10^5^/mL (B and D-F) or 1.2 × 10^5^/mL (3 × 10^4^/well) and 6 × 10^5^/mL (1.5 × 10^5^/well) (C), were cultured with CpG-matured, peptide-loaded cDC at a ratio of cDC:CD8 = 1:10 for 4 days. Divided CD8 cells were sorted by flow cytometry and 1 × 10^6^ (B; D-F) or 2 × 10^4^ (C) cells were adoptively transferred into vaccinia virus-infected C57BL/6J mice (4 days post-infection). (A) Outline of the experimental scheme. (B and C) The number of TCR Vβ11^+^ F5 CD8 T cell was determined in the blood on day 8 (B) and in the spleen on day 32 after activation (C). (D) The expression of CD62L, NKG2D, CD29, CD49d, and CD49a was analyzed on TCR Vβ11^+^ F5 CD8 T cells from spleen on day 32 and represented as histogram. Representative histograms (top) and individual percentages of positive cells (bottom) are shown. (E) The expression of CD43 and CD27 was analyzed on TCR Vβ11^+^ F5 CD8 T cells from spleen on day 32. Representative histograms (left) and individual percentages of CD27^+^CD43^−^ and CD27^+^CD43^+^ cells (right) are shown. (F) On day 32, 3 × 10^6^ splenocytes were stimulated with NP68 (10 nM) for 4 h. The expression of IFN-γ and CCL5 by F5 CD8 T was analyzed by flow cytometry. One representative out of four independent experiments is presented. The results are expressed as the mean ± SD (n = 4 mice per group). The statistical significance of differences was determined by the Student’s *t* test (ns = p > 0.05, ∗ = p ≤ 0.05).

To evaluate the capacity of *in vitro*-activated CD8 T cells to participate in the antiviral immune response and therefore, to differentiate into effector and memory cells, we sorted F5 CD8 T cells after 4 days of activation in the presence or absence of ex-IL-2 and transferred them into C57BL/6J mice challenged with vaccinia virus (VV) 4 days earlier (Figure 3A). On day 8 post-activation, similar numbers of effector cells were recovered in the blood, whether cells had been activated with or without ex-IL-2 (Figure 3B). Similarly, after 32 days, F5 CD8 T cells activated with or without ex-IL-2 gave rise to similar numbers of memory CD8 T cells (Figure 3C). This was even true when cells were cultured at low density, a condition where they depend more on ex-IL-2 (Figure 2). Next, we analyzed the phenotype of the memory cells generated. To do so, we transferred a higher number of *in vitro*-activated F5 cells. The presence of ex-IL-2 during priming did not influence the expression of NKG2D or integrins essential for tissue migration such as CD29 and CD49a, and slightly affected the expression of CD62L and CD49d (Figures 3D and S3B). The patterns of CD27 and CD43 expression, that defines three distinct subpopulations of memory CD8 T cells that differ significantly in their recall response capacities to a viral lung infection,^15^ was analyzed. Following both priming conditions, memory cells predominantly adopted a CD27^+^CD43^−^ phenotype, which was found to be associated with better recall responses^15^ (Figures 3E and S3B). Finally, F5 memory cells restimulated with NP68 peptide produced similar levels of IFN-γ and CCL5 whether ex-IL-2 was added to the *in vitro* cultures or not (Figure 3F). In summary, these results indicate that cells activated with and without ex-IL-2 have a similar potential to participate in a primary immune response and differentiate into memory cells after transfer into infected hosts.

### When transferred into naive hosts, CD8 T cells activated in the presence of ex-IL-2 give rise to more memory cells that harbor increased memory traits

**Figure 4:**
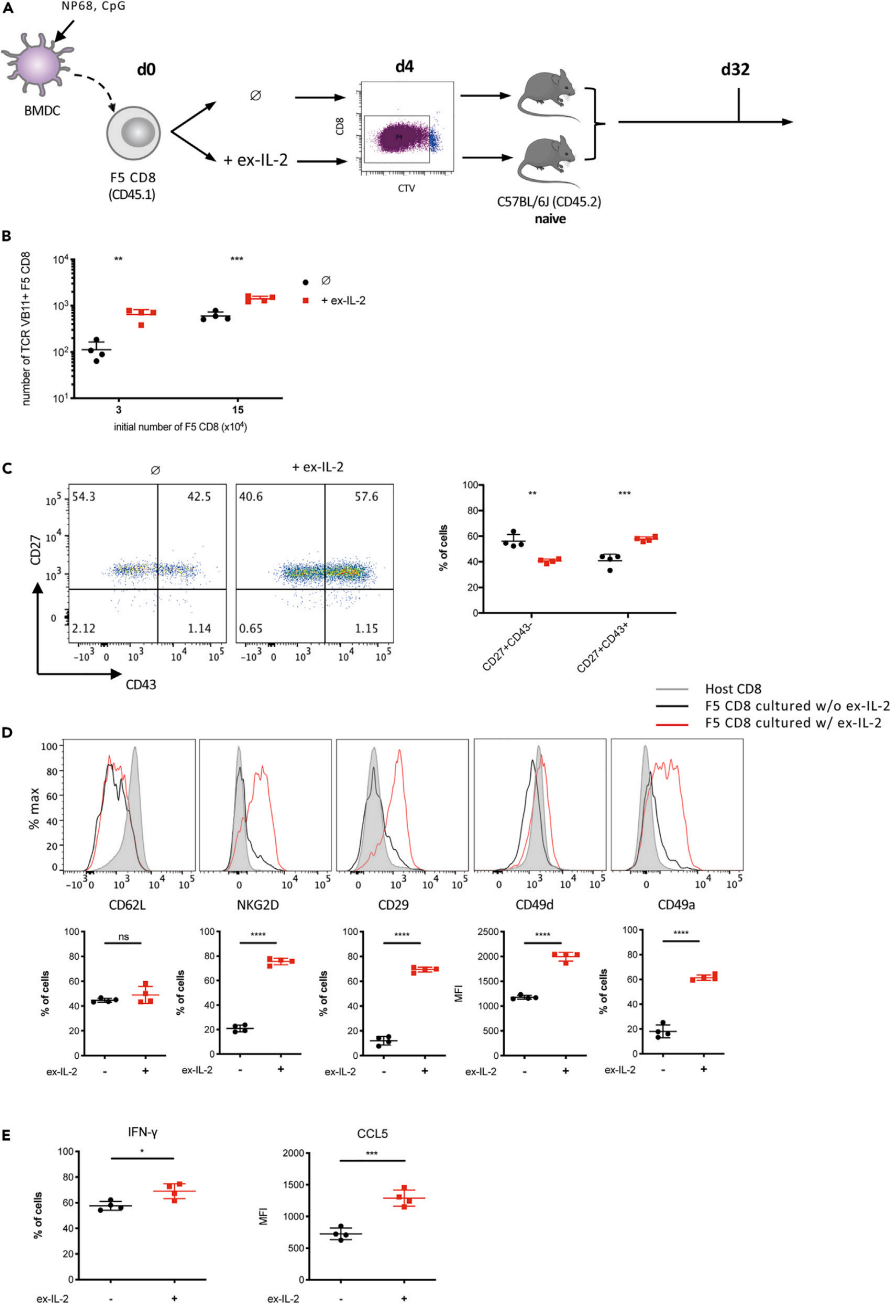
Ex-IL-2 promotes direct *in vivo* memory differentiation of *in vitro* activated cells See also Figure S3. (A–E) CTV-labelled purified naive F5 CD8 T cells at a concentration of 6x10^5^/mL (C-E) or 1.2 × 10^5^/mL (3 × 10^4^/well) and 6 × 10^5^/mL (1.5 × 10^5^/well) (B) were cultured with CpG-matured, peptide-loaded cDC at a ratio of cDC:CD8 = 1:10 for 4 days. Divided CD8 cells were sorted by flow cytometry and 1 × 10^6^ (C-E) or 2 × 10^4^ (B) cells were adoptively transferred into naive C57BL/6J mice. (A) Outline of the experimental scheme. (B) The number of TCR Vβ11^+^ F5 CD8 T cell was determined in the spleen on day 32 after activation. (C) The expression of CD43 and CD27 was analyzed on TCR Vβ11^+^ F5 CD8 T cells from spleen on day 32. Representative histograms and individual percentages of CD27^+^CD43^−^ and CD27^+^CD43^+^ cells are depicted. (D) The expression of CD62L, NKG2D, CD29, CD49d and CD49a was analyzed on TCR Vβ11^+^ F5 CD8 T cells from spleen on day 32. Representative histograms and individual values for each mouse are shown. (E) On day 32, 3 × 10^6^ splenocytes were stimulated with NP68 (10 nM) for 4 h. The expression of IFN-γ and CCL5 by F5 CD8 T was analyzed by flow cytometry. One representative out of four independent experiments is presented. The results are expressed as the mean ± SD (n = 4 mice per group). The statistical significance of differences was determined by the Student’s *t* test (ns = p > 0.05, ∗ = p ≤ 0.05, ∗∗ = p ≤ 0.01, ∗∗∗ = p ≤ 0.001, ∗∗∗∗ = p ≤ 0.0001).

To further explore the impact of ex-IL-2 during the primary response on memory cells generation, we next adoptively transferred F5 CD8 T cells activated with or without ex-IL-2 for 4 days into naive C57BL/6J mice (Figure 4A). In this context, *in vitro* activated CD8 T cells are not further stimulated by infection-associated inflammation or antigen and only receive homeostatic survival signals. The number and phenotype of F5 CD8 T cells recovered from the spleen were analyzed 32 days post-activation. We found that the addition of ex-IL-2 during priming increased the number of resulting memory cells whether initially activated at low or high density (Figure 4B). Cells activated with ex-IL-2 differentiate more into CD27^+^ CD43^+^ memory cells, whereas they tend to adopt a CD27^+^CD43^−^ phenotype in the absence of ex-IL-2 during priming (Figures 4C and S3C). Furthermore, the presence of ex-IL-2 during priming resulted in higher expression of CD29, CD49a, CD49d and NKG2D (Figures 4D and S3C) but did not impact CD62L expression. Finally, cells activated with ex-IL-2 produced more IFN-γ and expressed higher level of CCL5 following NP68 restimulation (Figure 4E). Overall, these results showed that the presence of ex-IL-2 during *in vitro* priming promotes, after transfer into naive hosts, the generation of more memory CD8 T cells that display increased memory traits.

### Single-cell RNA-sequencing analysis reveals that ex-IL-2 delays the differentiation of activated CD8 T cells into memory precursor while driving their acquisition of effector functions

**Figure 5:**
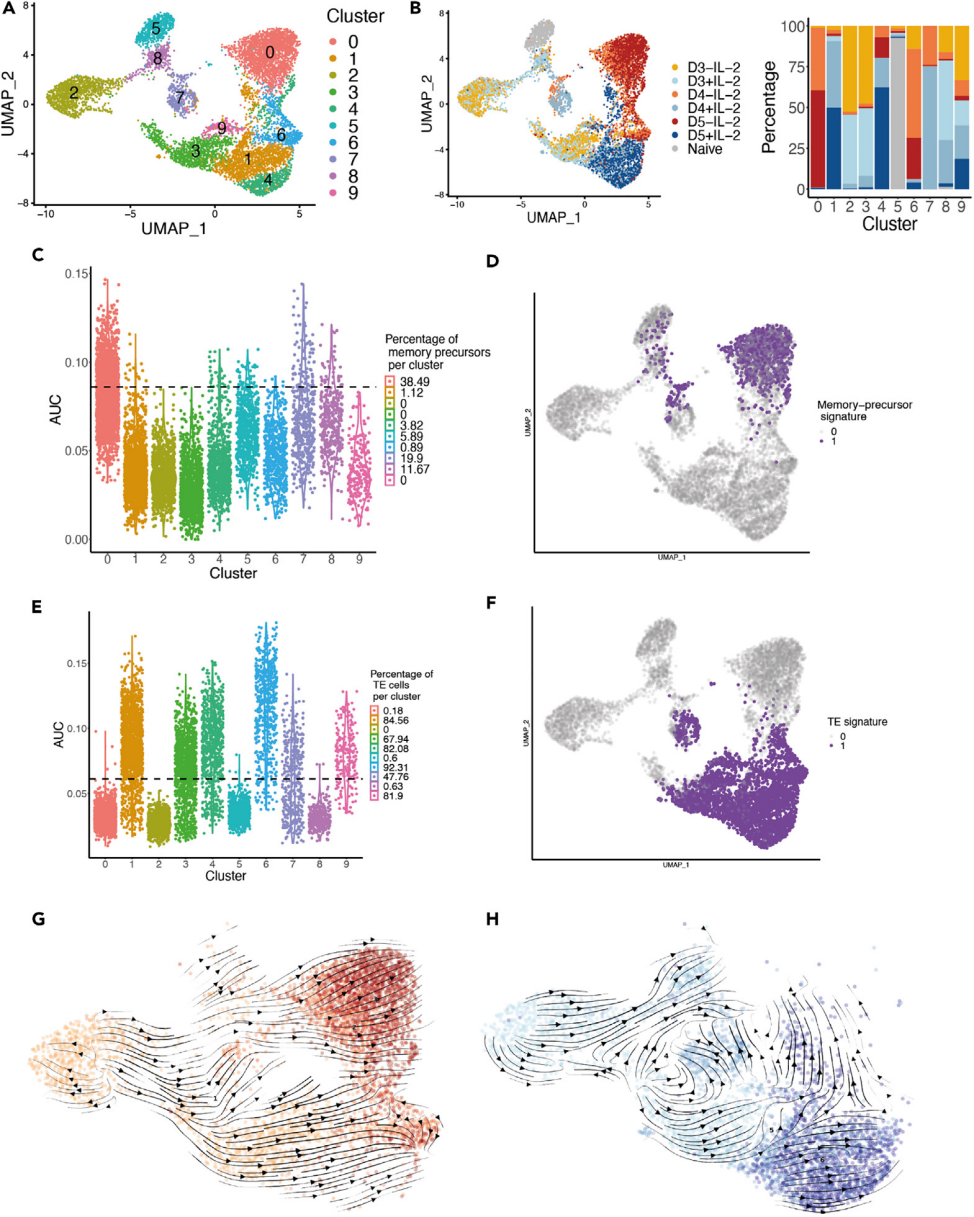
Single cell RNA seq analysis reveals that ex-IL-2 delays the differentiation of activated CD8 T cells in memory precursor, while driving the acquisition of effector function See also Figures S4 and S5. (A) UMAP projection of cells sorted on day 0, 3, 4 and 5 after activation with or without ex-IL-2 and colored according to cluster identification by Seurat package. (B) **Left:** UMAP projection of cells as in A, colored according to the experimental time points and conditions of culture (+/− ex-IL-2). **Right:** Percentages of cells from each experimental condition in each cluster. (C and E) Memory precursor (C) and terminal effector (TE) (E) signature enrichment per cluster. The dotted line represents the threshold above which cells are considered positive for the gene expression signature. The legend indicates the percentage of cells positive for a given signature in each cluster. AUC: area under the curve. (D and F) The cells positive for the memory precursor (D) or the terminal effector (TE) (F) signature are colored on the UMAP. (G and H) RNA velocities of cells activated without ex-IL-2 (G) or with ex-IL-2 (H) for 3, 4 or 5 days are projected onto the UMAP. Colors are the same as in **B**.

To investigate the transcriptional differences and changes that ex-IL-2 could mediate over the course of *in vitro* activation, we performed scRNA-seq on F5 CD8 T cells activated with or without ex-IL-2. Cells were collected 3, 4 or 5 days after activation and naive cells were sorted as a control. Clustering analysis partitioned cells into 10 clusters that can be visualized on a UMAP dimensional reduction (Figure 5A). Cells collected on day 3, whether grown with or without ex-IL-2, were partitioned in similar proportion between cluster 2 and 3, indicating a similar gene expression pattern (Figure 5B). Interestingly, cells from cluster 2 displayed an enrichment in genes related to regulation of growth and apoptosis (Gadd45b, Ddit3) and were, based on their gene expression, associated with the G1 phase of the cell cycle. In contrast, cells from cluster 3 expressed genes associated with early activation and differentiation (Srm, Ybx3), and cell cycle (Tuba4a, Pclaf, Top2a) and were likely in cycle (Figures S4A–S4C). The transcriptional programs of CD8 T cells activated with and without ex-IL-2 diverged from day 4 onwards with the majority of CD8 cells activated with ex-IL-2 accumulating in cluster 1 and 4, while cells activated without ex-IL-2 where mainly found in cluster 6 and 0 (Figures 5A, 5B, and S5). We observed that cells from clusters 1 and 4 expressed genes associated with cell division (Top2a, Pclaf, Mki67) and were classified as being in cycle (Figures S4A–S4C). Conversely, cells in cluster 0 completely downregulated these genes and were classified as cells in G1, indicating that in the absence of ex-IL-2, CD8 T cells acquired a quiescent state more rapidly. We next performed a gene set enrichment analysis (GSEA) using the MPs gene signature from Yao et al.^16^ and a terminal effector (TE) signature from Kanbar et al.^17^ The MP signature was expressed by a large fraction (approximately 40%) of the cells in cluster 0 (Figures 5C and 5D) while the effector signature showed a strong enrichment in cells from clusters 1, 3, 4, 6, 7 and 9 (Figures 5E and 5F). Cells from cluster 3 and 6 contained a significant fraction of CD8 T cells activated in the absence of ex-IL-2 (Figure 5B), suggesting that a fraction of these cells develop effector functions. Our previous results suggested that following activation, MP cells could derive from bipotent cells that retain MP potential while developing effector functions.^18^ In order to identify cells with such a profile, we searched for cells that expressed the combined MP and TE signatures. Clusters 1, 4 and 6 were enriched in cells expressing both signature, with cluster 6 containing the largest fraction (about 70%) and cluster 1 and 4 containing a significant fraction with about 24% of positive cells in each cluster (Figures S4D and S4E). To further define the dynamics of cell differentiation between the different clusters, the differentiation trajectories of cells were determined by applying the scVelo algorithm^19^ and were projected onto the UMAP representation (Figure S4F). This analysis revealed two cell trajectories, with a first trajectory of differentiation starting from day 3 cells in cluster 2, passing through cluster 3 and leading to clusters 1 and 4 which contain cells activated in the presence of ex-IL-2. The second trajectory of differentiation also started from cluster 2, passing through cluster 8 and finishing in cluster 0, that contained CD8 T cells activated without ex-IL-2 and was enriched in cells expressing the MP signature (Figure S4F). The scVelo trajectory projection corresponds to the average RNA velocities of cells within a given location/cluster. To determine if cells activated with or without ex-IL-2 had the same dynamics along the trajectories, we projected their RNA velocity trajectories independently on the UMAP (Figures 5G and 5H). Interestingly, CD8 T cells activated in the absence of ex-IL-2 could follow two paths from day 3 onwards: (i) a small fraction of those cells became effector (clusters 3, 1 and 4) with some of them then moving toward MP cells while (ii) a larger fraction directly moved toward quiescent MP cells (cluster 0) (Figure 5G). Conversely, CD8 T cells activated in the presence of ex-IL-2 mainly followed an effector differentiation trajectory toward cluster 1 and 4 with a few cells in cluster 1, 4 and 6 being directed toward cluster 0 that is enriched in MP cells (Figure 5H).

In summary, in the absence of ex-IL-2, CD8 T cells start acquiring quiescent MP traits from 4 days in culture while in the presence of ex-IL-2 they instead strongly adopt an effector transcriptional program.

### CD8 T cells activated in the presence of ex-IL-2 display stronger effector functions

**Figure 6:**
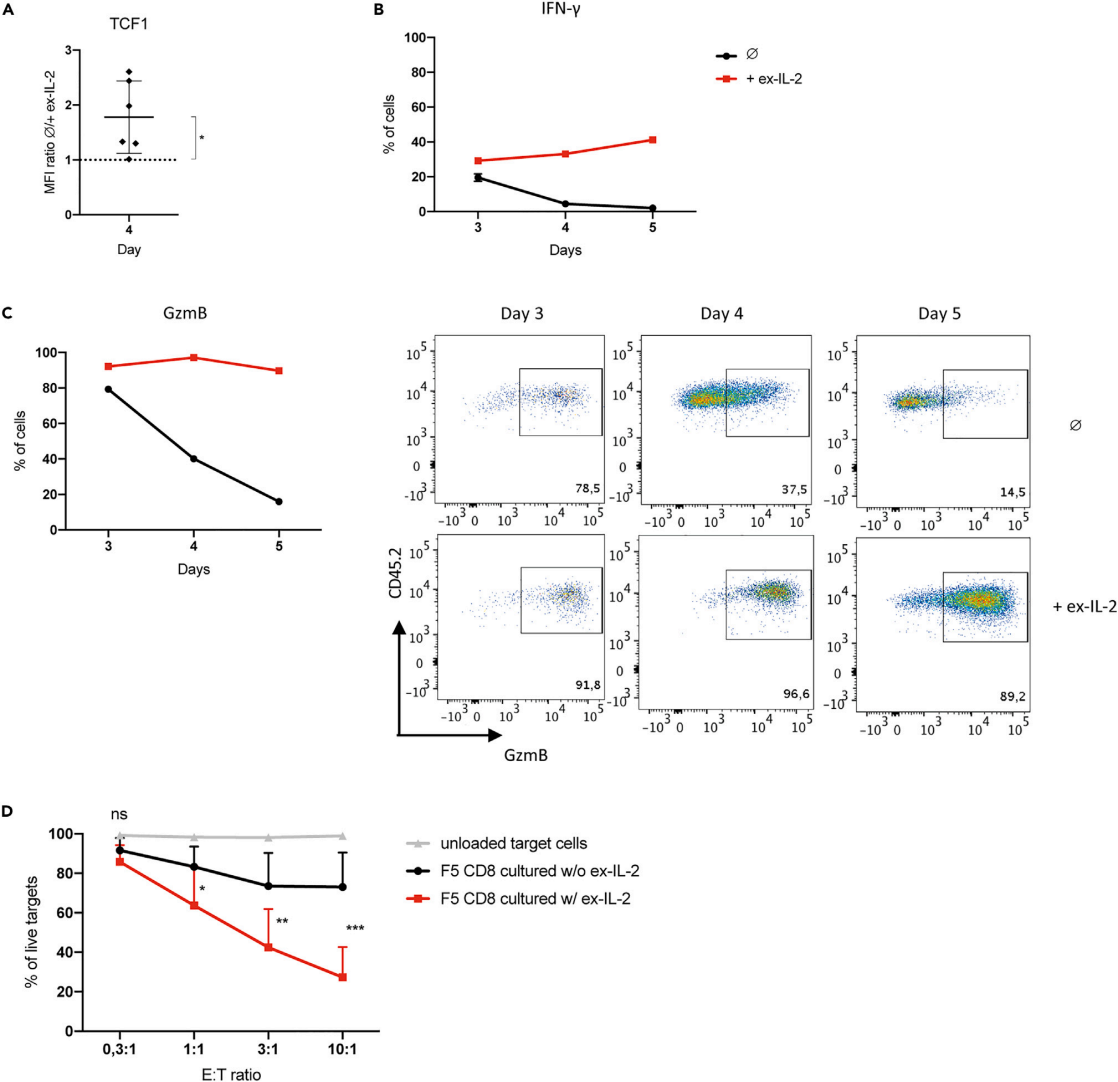
CD8^+^ T cells activated in the presence of ex-IL-2 display stronger effector functions See also Figure S6. 1.5 × 10^5^ magnetically purified naive F5 CD8^+^ T cells labeled with CTV were activated with CpG-matured, NP68-loaded cDC at a ratio of cDC:CD8 = 1:10, in the presence or absence of ex-IL-2 (11,5 ng/mL). (A) Median Fluorescence Intensity (MFI) of TCF1 was measured on divided cells after 4 days of activation and the ratio between cells cultured in the absence and cells cultured in the presence of ex-IL-2 was calculated. (B and C) After 3, 4, or 5 days of activation, CD8^+^ T cells were restimulated for 2h with NP68 and the percentages of IFN-γ- (B) and GzmB- (C Left) expressing CD8^+^ T cells were measured on divided cells. Representative dot plots and individual percentages of GzmB^+^ cells are also depicted (C Right). (D) Percentages of live EL4 target cells, loaded or not with NP68 peptide, after 4h of co-culture with effector CD8^+^ T cells activated for 5 days. Values from three independent experiments are presented. The results are expressed as the mean +SD. E:T ratio = effector:target ratio. The mean ± SD of six independent experiments is shown in panel A. The mean ± SEM of triplicate cultures from one representative experiment out of three independent experiments is shown in panel B and C (right). In A, the statistical significance of the difference between the mean value of ratios and the hypothetical value of 1 was determined by the one sample t-test. In D, the statistical significance of differences between F5 CD8^+^ cultured with or without ex-IL-2 at each E:T ratio was assessed by a two-way ANOVA followed by Sidak’s multiple comparison test (ns = p > 0.05, ∗∗ = p ≤ 0.01 and ∗∗∗ = p ≤ 0.001).

To confirm the impact of ex-IL-2 on the differentiation trajectories of CD8 T cells following their activation, we measured the expression of several proteins associated with the MP or TE signatures after 3, 4 or 5 days of activation. The transcription factor TCF1, encoded by Tcf7, is a strong regulator of CD8 differentiation known to be highly expressed in naive and memory cells, but downregulated in terminal effector cells.^20^^,^^21^ In accordance with what has been observed in our scRNA-seq experiments, cells activated in the absence of ex-IL-2 kept a high level of TCF1 expression after 4 days, whereas it was decreased in the presence of ex-IL-2 (Figure 6A). Conversely, proteins associated with effector functions such as IFN-γ or GzmB were expressed by a huge proportion of CD8 T cells in the presence of ex-IL-2, whereas this proportion falls from day 4 onwards in its absence (Figures 6B, 6C, and S6). We thus assessed the ability of these cells to specifically kill target cells after 5 days of activation. To this end, we loaded EL4 cells with the NP68 peptide and co-cultured them at different effector:target (E:T) ratios. As expected, F5 CD8 T cells activated in the presence of ex-IL-2 turned out to be significantly more efficient in inducing the death of target cells (Figure 6D). These findings validate the impact of ex-IL-2 on the fate of CD8 T cells that was suggested by our scRNA-seq results. From day 4 onwards, cells activated in the presence of ex-IL-2 largely expressed effector proteins and showed greater cytotoxic capacities whereas in its absence, cells expressed a key transcription factor associated with memory cell differentiation.

## Discussion

IL-2 is a growth factor driving the proliferation and expansion of T cells.^22^^,^^23^^,^^24^ In this study, we showed that ex-IL-2 was dispensable during the initial TCR-induced CD8 T proliferation *in vitro* when cells were cultured at a high density, very probably owing to autocrine IL-2 secretion. However, when cells were cultured at low density, addition of ex-IL-2 was associated with a better CD8 expansion. This fits with the quorum sensing capacity of CD8 T cells recently described by Zenke et al., who showed that CD8 T cells communicate with each other and change their behavior in a population-dependent manner.^25^ Cellular cooperation could also take place between activated CD8 T cells and other cells as the addition of spleen cells to the culture led to increased CD8 T cell proliferation. Further studies are needed to define the mechanisms involved in this process. Moreover, we observed a better differentiation toward a central memory phenotype when CD8 T cells are cultured at higher density, similarly to what has been described for CD4 T cells.^14^

Several studies have suggested that the initial encounter and stimulation by antigen is sufficient to drive the proliferation and the differentiation program leading to the generation of effector and memory CD8 T cells.^1^^,^^26^^,^^27^^,^^28^^,^^29^ Naive CD8 T cells that have been activated for a brief period could continue to expand and differentiate in the absence of further stimulation in an autopilot process.^29^ Here, we showed that CD8 T cells activated in the absence of ex-IL-2 indeed proliferated *in vitro* to a similar extent as cells activated with ex-IL-2. This is in agreement with previously published data showing that *in vivo*, IL-2 knockout and IL-2Rα knockout CD8 T cells performed the same number of divisions compared to wild-type CD8 T cells in a context of viral or tumoral immunization.^30^ Importantly, F5 CD8 T cells activated in the absence of ex-IL-2 for 4 days remain functional and able to participate to an ongoing immune response against vaccinia virus expressing NP68, when transferred *in vivo* into infected murine hosts. Furthermore, they gave rise to memory CD8 T cells with identical phenotypes and function as compared to cells activated in the presence of ex-IL-2.

Although the presence of ex-IL-2 does not affect the number of divisions performed by CD8 T cells*,* we observed an increased expression of IL-2-target genes such as CD25 and Bcl-2 and sustained phosphorylation of STAT5 and Akt when CD8 T cells were cultured in the presence of ex-IL-2. This indicated that cells responded to ex-IL-2 and that this interaction led to the sustained development of the effector CD8 program. This is in agreement with the results of Toumi et al., showing that paracrine IL-2 was able to induce CD8 T cells differentiation into effector cells.^10^ In contrast, the IL-2 produced by activated cells in the absence of ex-IL-2 was not sufficient to maintain CD25 expression as well as the activation of its downstream signaling pathways. In this context, activated CD8 T cells rapidly adopted a memory phenotype gene expression signature and were able to differentiate into memory cells when transferred into a naive host. This is in agreement with the study by Kahan et al., showing that the fraction of CD8 T cells capable of producing IL-2 had a reduced ability to activate the STAT5 pathway and adopted a memory phenotype more rapidly.^4^

Our single-cell transcriptomic analyses of CD8 T cells activated *in vitro* indicated that in the presence of ex-IL-2, from day 4, most of the cells were cycling and positive for an effector cells gene expression signature. We confirmed that cells cultured with ex-IL-2 expressed more IFN-γ and GzmB and were more potent in killing target cells. Conversely in the absence of ex-IL-2, although cells can go through an effector stage on day 3, a large fraction of CD8 T cells directly acquired a quiescent MP gene expression signature without developing a full effector genes panel. Moreover, in naive hosts *in vivo*, they gave rise to memory cells with few memory effector traits. This is in contrast with the phenotype of memory cells generated from cells activated with ex-IL-2, that showed an increased ability to produce the effector cytokines IFN-γ and CCL5. Moreover, those cells expressed the antigen-induced associated marker NKG2D, and high levels of integrins important for cell homing to lung tissue such as CD29, CD49a and CD49d.^31^ Notably, more memory cells were recovered following adoptive transfer into a naive host when CD8 T cells were activated in the presence of ex-IL-2. This could be due in part to the increased Bcl-2 expression that is observed in the presence of ex-IL-2.

Importantly, these results indicate that the presence of ex-IL-2 during the *in vitro* activation did not negatively impact the capacity of CD8 T cells to differentiate into memory cells *in vivo*. This is in contrast to previously published work showing that IL-2 could inhibit this capacity. However, the concentration of IL-2 used was at least 50-fold higher than the one used here.^7^

Thus, the addition of IL-2 mimicking a paracrine signal promotes effector differentiation *in vitro* as it has been demonstrated *in vivo.*^4^^,^^5^^,^^10^ This function of IL-2 is not restricted to naive cells as in a chronic infection or tumor context, IL-2 signaling combined with anti-PD1 immunotherapy drove the differentiation of exhausted stem-like progenitors CD8 T cells toward a population with increased effector functions and enhanced antiviral and antitumor responses,^32^^,^^33^ thereby highlighting the therapeutic potential of IL-2 in the treatment of cancer and chronic infections.

Our results could also help to improve the generation of therapeutic T cells. Indeed, T-cell-transfer-therapies such as CAR-T cells (chimeric antigen receptor–T cell) have been a remarkable achievement in anticancer immunotherapy. The generation of these cells from patient T cells requires their activation and expansion *ex vivo*. This is usually done by activating cells in culture with anti-CD3 antibodies, supplemented with IL-2 to support their expansion. The robust proliferation and survival of therapeutic T cells *in vivo* are regarded as critical indicators of the clinical response in patients with B-cell malignancies and solid tumors.^34^^,^^35^^,^^36^ However, a significant fraction of patients relapses after immunotherapy or is refractory to the treatment. The quality of T cells that are transferred is one of the limiting factors that has been identified.^37^ Experiments in preclinical models have shown that the degree of differentiation of CD8 T cells used to prepare the therapeutic T cells inversely correlated with the magnitude of tumor rejection.^37^^,^^38^^,^^39^ The cytokines used to support *in vitro* T cell proliferation also influence the quality of T cells that are generated.^40^ Hence, one of the potential improvements in the production of therapeutic T cells would be to reassess the role of IL-2 in the generation of T cells that are efficient in killing tumor cells and that will persist as memory cells once transferred *in vivo*.

In conclusion, our results support a model in which ex-IL-2 is redundant for the initial CD8 T cell expansion but is essential to support the acquisition of effector functions. Furthermore, ex-IL-2 delays, but does not abrogate, the generation of MP cells, allowing them to imprint strong effector traits.

### Limitation of the study

Our study suggests that exposure to exogenous IL-2 delays the development of MP cells and promotes the acquisition of an effector program that is more strongly imprinted in memory cells generated in the presence of ex-IL-2. In this study, we only looked at the expression of integrins and cytokines by memory cells cultured in presence or absence of ex-IL-2 after *in vitro* restimulation. It could be of interest to adoptively transfer equal numbers of purified memory CD8 T cells, generated *in vivo* from CD8 activated *in vitro* with or without ex-IL-2, into naive mice prior to VV-NP68 challenge to evaluate their recall expansion potential and polyfunctionality. However, the low number of memory cells recovered was a limiting point.

## STAR★Methods

### Key resources table

**Table:** 

REAGENT or RESOURCE	SOURCE	IDENTIFIER
**Antibodies**		

BUV395 anti-mouse CD8a (Clone 53-6.7)	BD Biosciences	Cat#563786 (AB_2732919)
BV510 anti-mouse CD62L (MEL-14)	BD Biosciences	Cat#563117 (AB_2738013)
BUV737 anti-mouse CD45.1 (clone A20)	BD Biosciences	Cat#564574 (AB_2738850)
BV786 anti-mouse CD45.2 (clone 104)	BD Biosciences	Cat#563686 (AB_2738375)
PerCP-Cy5.5 anti-mouse CD11b (clone M1/70)	BD Biosciences	Cat#550993 (AB_394002)
PerCP-Cy5.5 anti-mouse CD11c (clone HL3)	BD Biosciences	Cat#560584 (AB_1727422)
PerCP-Cy5.5 anti-mouse CD19 (clone 1D3)	BD Biosciences	Cat#551001 (AB_394004)
PE anti-mouse TCF1 (clone S33-966)	BD Biosciences	Cat#564217 (AB_2687845)
PE-CF594 anti-AKT (clone M89-61)	BD Biosciences	Cat#562465 (AB_2737620)
BV650 anti-rat/mouse CD49a (clone Ha31/8)	BD Biosciences	Cat#740519 (AB_2740235)
BV711 anti-mouse CD314 (NKG2D) (clone CX5)	BD Biosciences	Cat#563694 (AB_2722498)
Biotin anti-mouse TCR Vβ11 (clone RR3-15)	BD Biosciences	Cat#553196 (AB_394702)
PE-Cy7 anti-mouse IFN-γ (clone XMG1.2)	BD Biosciences	Cat# 557649 (AB_396766)
Alexa Fluor 700 anti-mouse CD25 (clone PC61)	Biolegend	Cat#102024 (AB_493709)
FITC anti-rat/mouse-Bcl-2 (clone BCL/10C4)	Biolegend	Cat#633504 (AB_2028394)
BV605 anti-mouse/human CD44 (clone IM7)	Biolegend	Cat#103047 (AB_2562451)
PE anti-mouse CCL5 (clone 2E9)	Biolegend	Cat#149103 (AB_2564405)
PE-Cy7 anti-mouse CD43 (clone 1B11)	Biolegend	Cat#121218 (AB_528813)
PE anti-mouse Granzyme B (clone NGZB)	eBioscience	Cat#12-8898-82 (AB_10870787)
FITC anti-mouse phospho-STAT5 (clone SRBCZX)	eBioscience	Cat#11-9010-42 (AB_2572520)
PerCP-eFluor 710 anti-mouse EOMES (clone Dan11mag)	eBioscience	Cat#46-4875-82 (AB_10597455)
PE-Cy7 anti-mouse CD29 (clone eBioHMb1-1)	eBioscience	Cat#25-0291-82 (AB_1234962)
FITC anti-mouse CD27 (clone LG.7F9)	eBioscience	Cat#11-0271-82 (AB_465001)
PerCP-eFluor 710 anti-mouse CD49d (clone R1-2)	eBioscience	Cat#46-0492-82 (AB_11150051)

**Bacterial and virus strains**		

VV-NP68	Dr. D.Y.-L. Teoh (Human Immunology Unit, Institute of Molecular Medicine, Oxford, U.K.)	Modified from the Western Reserve strain

**Chemicals, peptides, and recombinant proteins**		

DMEM	Thermofisher	Cat#61965-026
RPMI	Thermofisher	Cat#61870-010
Sodium pyruvate (100mM)	Thermofisher	Cat#11360-039
HEPES (1M)	Thermofisher	Cat#15630-056
Gentamicin (50 mg/mL)	Thermofisher	Cat#15750-037
Beta-mercaptoethanol	Thermofisher	Cat#31350-010
L-glutamine	Thermofisher	Cat#A2916801
DPBS	Thermofisher	Cat#14190-094
FBS	BioWest	Cat#S1810-500 (Lot#S13439S1810)
NaN3 (CAS# 26628-22-8)	Sigma-Aldrich	Cat#S2002-500 (Lot#MKBX7529V)
efluor780-coupled Fixable Viability Dye	Invitrogen	Cat#65-0865-18
CellTrace^TM^ Violet	Invitrogen	Cat#C34557
Flt3L	Amgen	N/A
CpG ODN 1826	InvivoGen	Cat#tlrl-1826-1
anti-CD3/CD28 coated beads	Thermofisher	Cat#11452D
GolgiStop^TM^	BD biosciences	Cat#554724
Flow-count fluorospheres	Beckman Coulter	Cat#7547053
2-NBDG	Thermofisher	Cat#N13195
CellTracker Deep Red	Invitrogen	Cat#C34565
Zombie Green Fixable Viability dye	Biolegend	Cat#423112
NP68 (ASNENMDAM)	Proteogenix	N/A
Murine rIL-2	Dr F. Melchers, (Basel Institute of Immunology, Basel, Switzerland)	N/A
TAPI-2 acetate salt (CAS# 689284-12-6)	Sigma-Aldrich	Cat#SML0420

**Critical commercial assays**		

CD8a^+^ T cell isolation kit	Miltenyi Biotec	Cat#130-104-075
Foxp3/Transcription Factor Staining Buffer Set kit	eBioscience	Cat#00-5523-00
IL-2 ELISA MAX^TM^ standard Set mouse kit	Biolegend	Cat#431001
IFN- γ ELISA MAX^TM^ standard Set mouse kit	Biolegend	Cat#430801

**Deposited data**		

Raw and analyzed data	This paper	GEO: GSE237866

**Experimental models: Cell lines**		

EL4 lymphoma cell line (ATCC® TIB-39™)	ATCC	Cat#TIB-39

**Experimental models: Organisms/strains**		

Mouse: C57Bl6/J: C57BL/6J	Charles River	Strain code: 632
Mouse: F5: B6/J-Tg(CD2-TcraF5,CD2-TcrbF5)1Kio/Jmar	Prof. D. Kioussis (National Institute of Medical Research, London, U.K.)	N/A

**Software and algorithms**		

BD FACSDiva (v8.0) software	BD Biosciences	N/A
Flowjo (v10.7.1)	Flowjo software	N/A
Prism (v9.3.1)	Graphpad software	N/A
Cutadapt	Martin^41^	https://doi.org/10.14806/ej.17.1.200
Alevin fry pipeline	He et al.^42^	https://alevin-fry.readthedocs.io/en/latest/
Fishpond	Zhu et al.^43^	https://bioconductor.org/packages/release/bioc/html/fishpond.html
Seurat v4	Hao et al.^44^	https://satijalab.org/seurat/
AUCell	Aibar et al.^45^	https://bioconductor.org/packages/release/bioc/html/AUCell.html
ScVelo	Bergen et al.^19^	https://scvelo.readthedocs.io/

### Resource availability

#### Lead contact

Further information and requests for resources should be directed to and will be fulfilled by the lead contact, Jacqueline Marvel (jacqueline.marvel@inserm.fr).

#### Materials availability

This study did not generate new unique reagents.

#### Data and code availability

• Accessions for publicly-available datasets used in this study are described in previous publications and in the key resources table. The original data described in this paper are available in the GEO databank under the reference GSE237866.
• This paper does not report original code. Code used to generate figures is available upon reasonable request from the lead contact.
• Any additional information required to reanalyze the data reported in this paper is available from the lead contact upon request.

### Experimental model and study participant details

#### Mice

C57BL/6J (CD45.2) mice were purchased from Charles River Laboratories (L’Arbresle, France). C57BL/10-Tg (Cd2-TcraF5, CD2-TcrbF5)1Kio/AnuApb mice were provided by Prof. D. Kioussis (National Institute of Medical Research, London, U.K.) and backcrossed on CD45.1 C57BL/6 background^46^ to obtain F5 TCR [B6/J-Tg (CD2-TcraF5, CD2-TcrbF5)1Kio/Jmar] transgenic mice. The F5 TCR recognizes the NP68 peptide from influenza A virus (ASNENMDAM) in the context of H2-Db. Mice were bred and housed under SPF conditions in our animal facility (AniRA-PBES, Lyon, France). Females and males were used indifferently, generally between 8 and 12 week-old. All experiments were approved by our local ethics committee (CECCAPP, Lyon, France) and accreditations have been obtained from governmental agencies.

#### Virus and reagents

The recombinant vaccinia virus expressing the NP68 epitope (VV-NP68), was engineered from the Western Reserve strain by Dr. D.Y.-L. Teoh, in Prof. Sir Andrew McMichael’s laboratory at the Medical Research Council (Human Immunology Unit, Institute of Molecular Medicine, Oxford, U.K.).

Murine rIL-2 was produced using the myeloma clone X63-Ag8.653 cell lines transfected with the mouse IL-2 gene (kind gift from Dr F. Melchers, Basel Institute of Immunology, Basel, Switzerland).

Complete RPMI and DMEM mediums consist of RPMI or DMEM medium (Life Technologies) supplemented with 10 or 6% FCS respectively, 10 mM HEPES, 50 mg/mL gentamicin, 2 mM L-glutamine (Life Technologies).

#### Bone marrow-derived dendritic cells (BMDCs) cultures

Mice were sacrificed by cervical dislocation and bone marrow progenitors were washed out from bones (femurs and tibias). 2 × 10^6^ cells/mL were incubated in complete RPMI medium with 100 ng/mL Flt-3L (kindly provided by Amgen) in 6 wells-plates as described in de Brito et al.^47^ After 7 days, NP68 peptide (20 nM) with or without CpG ODN 1826 (2 μg/mL, InvivoGen) was added and cells were cultured overnight. The fraction of cDC (CD11c^+^, B220^+^) was measured by flow cytometry and was always >65%.

### Method details

#### *In vitro* stimulation

CD44^−^ naive CD8 T cells were magnetically isolated from splenocytes of F5 TCR transgenic or C57BL/6J mice by negative selection using a specific CD8a^+^ T cell isolation kit (Miltenyi Biotec, #130-104-075) and autoMACS Pro Separator, according to the manufacturer instructions. Anti-CD44-Biotine antibody (IM7.8.1, 1 μL/1.5 × 10^8^ cells) was added to the cocktail of biotin-conjugated monoclonal antibodies to remove memory-phenotype CD8 T cells. Naive CD8 T cells purity was analyzed on FACS LSR Fortessa 4L and was always >97%.

Purified CD8 T cells were stained with CTV (CellTrace Violet, 2.5 mM, Thermofisher) according to manufacturer’s instructions. 1.5 × 10^4^ to 1.5 × 10^5^ CTV labelled-naive CD8 T cells were cultured in 250 μL complete DMEM medium with respectively 1.5 × 10^3^ to 1,5 × 10^4^ NP68-loaded matured BMDCs (ratio cDC:CD8 = 1:10) for up to 6 days at 37°C in 96 wells-U bottom plates, in the presence or absence of 5% murine rIL-2 supernatant (corresponding to a final concentration of 11.5 ng/mL) to remove memory-phenotype CD8 T cells. In some experiments, 3 × 10^5^ C57BL/6J splenocytes were added or recombinant IL-2 (Miltenyi Biotec) was used.

For some adoptive transfer experiments, 1.8 × 10^7^ CTV labelled-F5 were activated with 1.8 × 10^6^ NP68-loaded matured BMDCs in the presence or absence of 5% murine rIL-2 supernatant in complete DMEM medium, in 30 mL T25 flasks for 4 days.

Naive CD8 T cells (CD44^−^) were isolated from C57BL/6J spleen and stained with CTV as described above. 1.5 × 10^5^ CTV labelled-naive CD8 T cells were cultured in 250 μL complete DMEM medium with anti-CD3/CD28 coated beads at a 1 bead: 4 CD8 T cell ratio for 4 days, at 37°C in 96 well plate-U bottom plates, in the presence or absence of 5% supernatant containing IL-2.

#### *In vivo* memory CD8 T cell generation and restimulation

Purified naive CD8 T cells were activated *in vitro* for 4 days. The divided cells were sorted by flow cytometry (FACS Aria I, BD Biosciences) according to their CD44 expression and CTV dilution (see Figure S1A). Purity after sorting was >98%. 1 × 10^6^ or 2 × 10^4^ sorted cells were adoptively transferred by intravenous injection (i.v.) in VV-NP68 infected mice or uninfected mice. For immunization, mice were first anesthetized with an intraperitoneal (i.p.) injection of Ketamine (1.5 mg)/Xylazine (0.3 mg) in 150 μL PBS (Phosphate Buffer Saline) and then the VV-NP68 (2 × 10^5^ PFU) was intranasally (i.n.) administrated in 20 μL of PBS. Blood was collected after 4 days. After 28 days, mice were sacrificed by cervical dislocation. Spleens were harvested, mechanically disrupted, and filtered through a sterile 100-μm nylon mesh filter (BD Biosciences). Single cell suspensions were then stained for flow cytometry analysis. F5 CD8 T cells were detected based on the joint expression of CD45.1 and TCR Vβ11.

For flow cytometry detection of cytokine production, 3 × 10^6^ splenocytes were incubated with 10 nM NP68 peptide for 4h at 37°C in the presence of GolgiStop (BD Biosciences), according to manufacturer’s instructions.

#### Flow cytometry

In order to count cells, 100 μL of Flow-count fluorospheres (Beckman Coulter) were added before staining steps. Cells were first stained with efluor780-coupled Fixable Viability Dye (Thermo Scientific) for 15 min at 4°C. Cells were then incubated with an Fc receptor blocking antibody (2.4G2 hybridoma supernatant) for 10 min at 4°C followed by surface staining for 30 min at 4°C with the appropriate mixture of mAbs diluted in staining buffer (PBS supplemented with 1% FCS [Life Technologies] and 0.09% NaN3 [Sigma-Aldrich]). For biotin-coupled antibody, a further streptavidin staining step was performed for 10 min at 4°C. In some experiments, cells were incubated in 100 μL RPMI containing 100 μM 2-NBDG (ThermoFisher) for 10 min at 37°C before staining, to analyze glucose uptake. Intracellular staining was performed using eBioscience Foxp3/Transcription Factor Staining Buffer Set kit (ThermoFisher) for the analysis of cytokines and transcription factors, or Lyse/Fix and PermIII buffers (BD Biosciences) for the analysis of phosphorylated proteins, according to manufacturers’ instructions.

For some experiments, TAPI-2 acetate salt (Sigma-Aldrich) was added at a final concentration of 20 μM to all solutions used during the staining process, in order to avoid CD62L shedding.

The following antibodies were used: CD8 (53.6.7), CD62L (MEL-14), CD45.1 (A20), CD45.2 (104), CD11b (M1/70), CD11c (HL3), CD19 (1D3), TCF-1 (S33-966), AKT (M89-61), CD49a (Ha31/8), NKG2D (CX5), TCR Vβ11 (RR3-15), IFN-γ (XMG1.2) from BD Biosciences, CD25 (PC61), CD44 (IM7.8.1), Bcl-2 (BCL/10C4), CCL5 (2E9), CD43 (1B11) from Biolegend, Granzyme B (NGZB), EOMES (Dan11mag), CD29 (eBioHMb1-1), CD27 (LG.7F9), CD49d (R1-2) and phospho-STAT5 (SRBCZX) from eBioscience.

All analyses were performed on a BD Biosciences FACS Fortessa and analyzed with FlowJo software 10.7.1 (Tree Star, Ashland, OR).

#### Cytokines production measurement (ELISA)

IFN-γ and IL-2 in culture supernatants were measured using IFN-γ or IL-2 ELISA MAX Standard Set mouse kit (Biolegend), according to manufacturer instructions.

#### Single cell sorting and RNA sequencing

The scRNA-seq library was generated using Chromium Next GEM Single Cell 3′ reagent v.3.1 with feature barcode technology (10X Genomics) according to the manufacturer’s protocol. Briefly, 5 × 10^4^ naive F5 CD8 T cells were activated *in vitro* for 3, 4 and 5 days in the presence or absence of 5% IL-2. Cells of each experimental conditions were FACS stained as mentioned in the previous section using the following antibodies for surface staining: CD8 (53.6.7), CD45.1 (A20), CD45.2 (104), CD11b (M1/70), CD11c (HL3), CD19 (1D3) from BD Biosciences and CD44 (IM7.8.1) from Biolegend. Cells were then barcoded with hashtag oligonucleotides (HTO) antibodies specific for each condition (TotalSeq-B – Biolegend) for 30 min on ice. After washing with FACS buffer, cells were sorted on a FACS Aria III cytometer (BD Biosciences). Sorted cells were immediately loaded into the Chromium Controller (10X genomics, Pleasanton, California) to generate single-cell gel beads-in-emulsion (GEM). After reverse transcription, GEM were disrupted, barcoded cDNA were amplified by PCR and then size-selected to separate 3′ gene expression and cell surface protein (HTO) library construction according to the manufacturer’s instructions. On one hand, the gene expression library was fragmented, end-repaired and sample indexes were added by PCR. On the other hand, sample indexes were added by PCR for the HTO library. The purified libraries were quantified using the Library Quantification Sample Kit Kapa (Illumina-Uni San Diego, California). Gene expression and HTO libraries were pooled in a 4:1 ratio and paired-end (2 × 150 pb) sequenced (375M reads) on a HiSeq X platform (Illumina) with 1% of PhiX.

#### Single cell RNA-seq preprocessing

Quality control of raw data was performed using FastQC. The TSO, poly-A sequences and low-quality bases were trimmed using cutadapt.^41^ Transcript expression quantification was performed using the alevin-fry pipeline^42^ and version M31 of GENCODE mouse genome and annotations. The gene/count matrix was generated with fishpond.^43^ Cells with more than 7% mitochondrial counts were filtered-out. The HTO was demultiplexed using the Seurat pipeline^44^ and the RNA counts were normalized using the *sctransform* function (v2).

#### Single-cell RNA-seq analysis

Using Seurat, a UMAP was generated based on the first 30 components of a principal component analysis and clusters were defined using a resolution of 0.45. Differential expression analysis was made using the Seurat function *FindAllMarkers*. Heatmap and dot plot were generated using the Seurat function DoHeatmap and DotPlot respectively. The Seurat R package was used to classify cells into G1, S or G2/M phases of the cell cycle. The classifier, relying on a list of genes from Tirosh et al.^48^ contains markers of the G2/M and S phase. A score was attributed to each cell with a certain probability to belong to the S or G2/M phases. Cells expressing no S, G2/M markers are assigned to the G1 class. The AUCell R package^45^ was used in order to identify cells with active gene signature. The memory precursor signature was downloaded from the Yao et al. paper^16^ and the T effector signature from the Kanbar et al. paper.^17^ To infer RNA velocities and predict cell-specific trajectories, scVelo was used^19^ in dynamical mode.

#### *In vitro* cytotoxicity assay

1,5.10^5^ purified naive F5 CD8 T cells were activated *in vitro* for 5 days, as previously described. EL4 cell line was used as target cells. To this end, EL4 cells were incubated with 100 nM NP68 peptide (1h30) and stained with CellTracker Deep Red (0.1 μM, Thermofischer) according to manufacturer’s instructions. Activated F5 CD8 T cells and EL4 cells were then co-cultured at different effector:target (E:T) ratios in complete DMEM medium for 4h (37°C) in round bottom 96 well plates. EL4 cells were stained with Zombie Green Fixable Viability Dye (Biolegend) and survival was measured by flow cytometry.

The EL4 lymphoma cell line was obtained from the American Type Culture Collection (ATCC) (Manassas, VA) and cultured in complete DMEM medium.

### Quantification and statistical analysis

#### Statistical analysis

Statistical analyses were performed using Graph-pad software Prism 5. Two tailed unpaired t-test, one sample t-test, and one-way and two-way ANOVA followed by Tukey’s and Sidak’s post-hoc tests, respectively, were used as indicated in the figure legends.
