# Supplementary material for: Exogenous IL-2 delays memory precursors generation and is essential for enhancing memory cells effector functions
Source: iScience. 2024 Mar 4;27(4):109411. doi: 10.1016/j.isci.2024.109411 (PMC10952031; doi:10.1016/j.isci.2024.109411)
Supplement: Document S1. Figures S1–S6 [file mmc1.pdf]

**iScience, Volume *27***

## Supplemental information

# Exogenous IL-2 delays memory precursors generation and is essential for enhancing memory cells effector functions

**Shaoying Wang, Margaux Prieux, Simon de Bernard, Maxence Dubois, Daphne Laubreton, Sophia Djebali, Manon Zala, Christophe Arpin, Laurent Genestier, Yann Leverrier, Olivier Gandrillon, Fabien Crauste, Wenzheng Jiang, and Jacqueline Marvel**

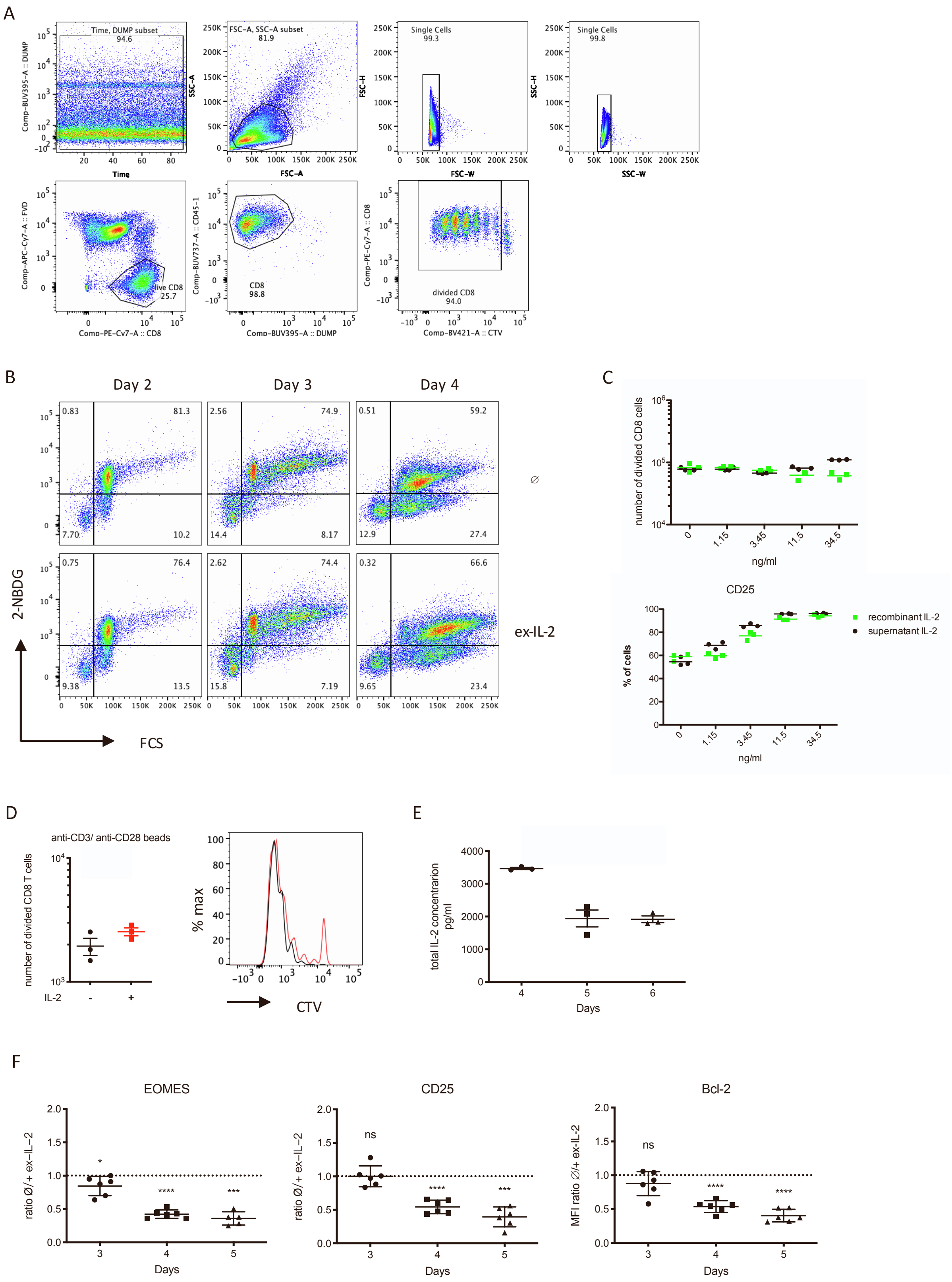


**Figure S1. Ex-IL-2 has no impact on glucose uptake or CD8 T cells proliferation but increases CD25 expression. Related to Figure 1.**

**A-B.** $1.5 \times 10^5$ magnetically purified naive F5 CD8 T cells labelled with CTV were cultured with CpG-matured, peptide-loaded cDC at a ratio of cDC:CD8 = 1:10, in the presence or absence of ex-IL-2.

**A.** Gating strategy of divided CD8 T cells is shown.

**B.** 2-NBDG (glucose uptake indicator) was analyzed on divided CD8. Representative dot plots are shown.

**C.** $1.5 \times 10^5$ magnetically purified naive F5 CD8 T cells labelled with CTV were cultured with CpG-matured, peptide-loaded cDC at a ratio of cDC:CD8 = 1:10, in the presence of various concentrations of supernatant-containing-IL2 or recombinant-IL-2 for 4 days. Equivalent concentrations of IL-2 were used (0.5, 1.5, 5 or 15% equivalent to 1.15, 3.45, 11.5 and 34.5 ng/ml, respectively). The number of divided CD8 T cells (top panel) and CD25 expression by divided CD8 T cells (bottom panel) are shown.

**D.** $1.5 \times 10^5$ magnetically purified naive C57BL/6J CD8 T cells labelled with CTV were cultured with anti-CD3/CD28 coated beads at a ratio of beads:CD8 = 1:4, in the presence or absence of ex-IL-2 for 4 days. The number of divided CD8 T cells (left panel) and CTV dilution in the presence (red) or absence (black) of 5% supernatant IL-2 are shown.

**E.** Secretion of IL-2 by CD8 T cells activated in the absence of ex-IL-2 as described in A-B was measured by ELISA in the culture supernatant at the indicated days.

**F.** Percentages of $EOMES^+$ and $CD25^+$ cells, as well as the Median Fluorescence Intensity (MFI) of Bcl-2 was measured on divided cells after 3, 4 or 5 days of activation. The ratio-cell-number or ratio-MFI for cells cultured in the absence or presence of ex-IL-2 was calculated.

The mean ± SEM of triplicate cultures from 1 experiment in C, and from 1 representative experiment out of 3 independent experiments in panel D-E, is presented. The mean ± SD of 6 independent experiments is shown in panel F, and the statistical significance of the difference between the mean value of ratios and the hypothetical value of 1 was determined by the one sample t-test (ns= $p>0.05$, ***= $p \leq 0.001$, ****= $p \leq 0.0001$).

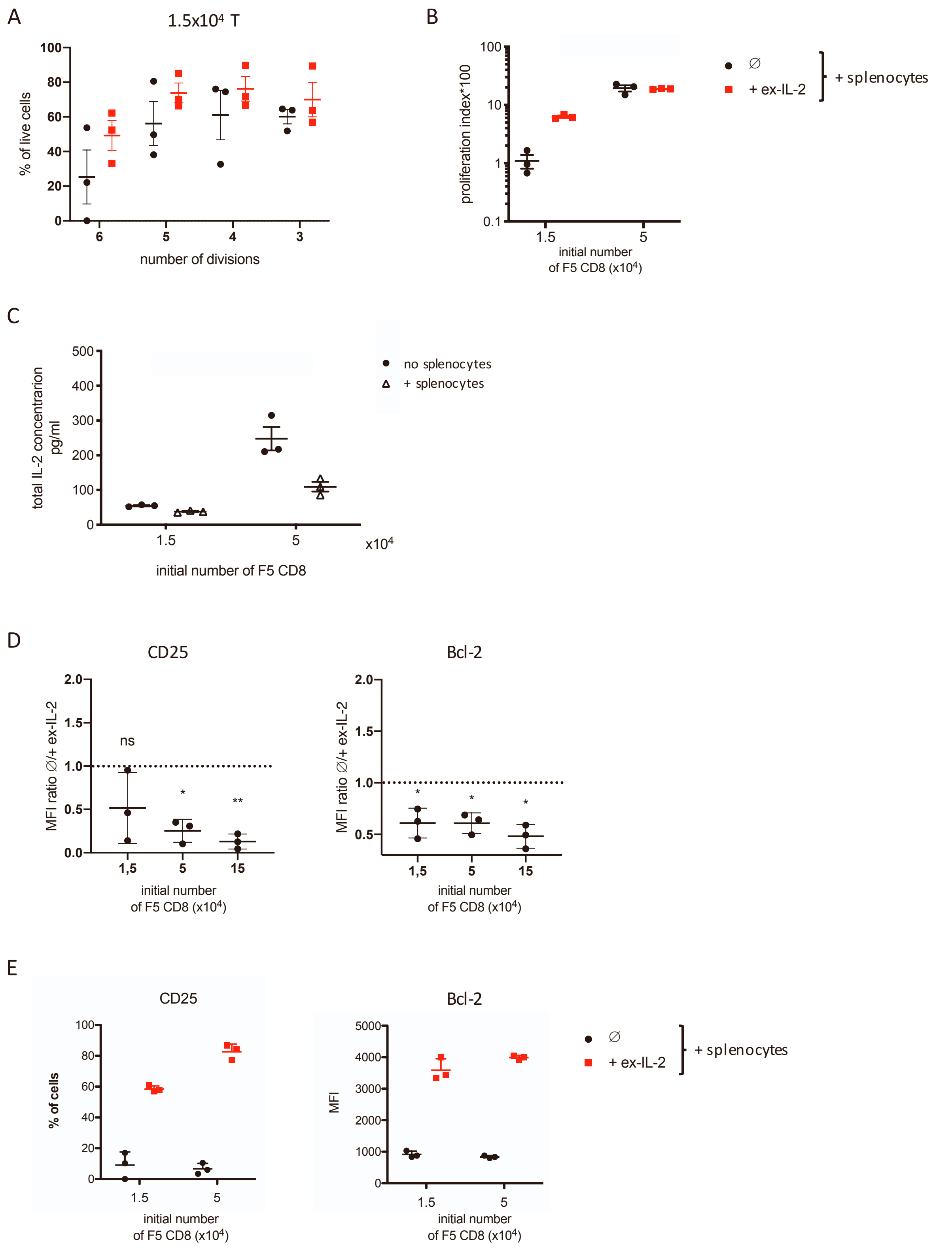


**Figure S2. Ex-IL-2 dependency is determined by the number of responding cells, rather than the number of total cells in the environment. Related to Figure 2.**

$1.5x10^4$, $5x10^4$ or $15x10^4$ magnetically purified naive F5 CD8 T cells labelled with CTV were cultured with CpG-matured, peptide-loaded cDC at a ratio of DC:CD8 = 1:10, in the presence or absence of ex-IL-2 (11,5 ng/ml) for 4 days. In B, C and E, $3x10^5$ splenocytes from C57BL/6J mice were added to sustain cell viability.

**A.** The percentage of live CD8 T cells was determined in each division peak for cells cultured at a density of $1.5x10^4$ in the absence or presence of ex-IL-2. Values from three independent experiments are presented. The results are expressed as the mean ± SD.

**B.** The number of divided cells was measured among activated CD8 T cells in the presence of $3x10^5$ C57BL/6J splenocytes to sustain cell viability and the proliferation index was determined as [(the number of divided cells/the initial number of CD8 T cells) *100].

**C.** Secretion of IL-2 by CD8 T cells activated in the absence of ex-IL-2 was measured by ELISA in the supernatant after 4 days, in the presence (empty triangles) or absence (full circles) of splenocytes.

**D.** Median Fluorescence Intensity (MFI) of CD25 and Bcl-2 was measured on divided cells for each cell concentration, and the ratio between cells cultured in the absence and cells cultured in the presence of ex-IL-2 was calculated.

**E.** Expression of CD25 and Bcl-2 by divided CD8 T cells activated in the presence of C57BL/6J splenocytes.

The mean ± SEM of triplicate cultures from 1 representative experiment out of 3 (A) or 2 (B, C and E) independent experiments, is presented. The mean ± SD of 3 independent experiments is shown in panel D, and the statistical significance of the difference between the median of ratio values and the hypothetical value of 1 was determined by the one sample t-test (ns= $p>0.05$, *= $p\leq0.05$, **= $p\leq0.01$).

A

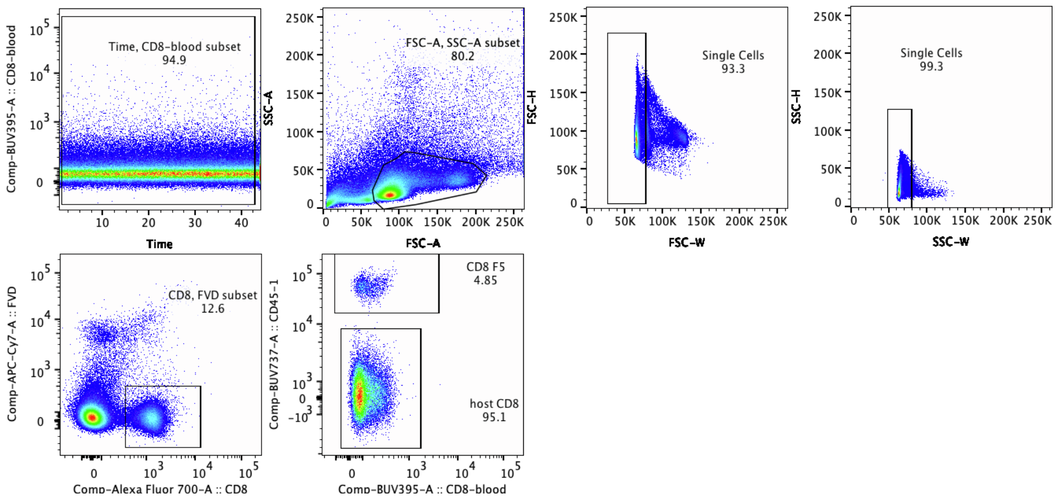


B

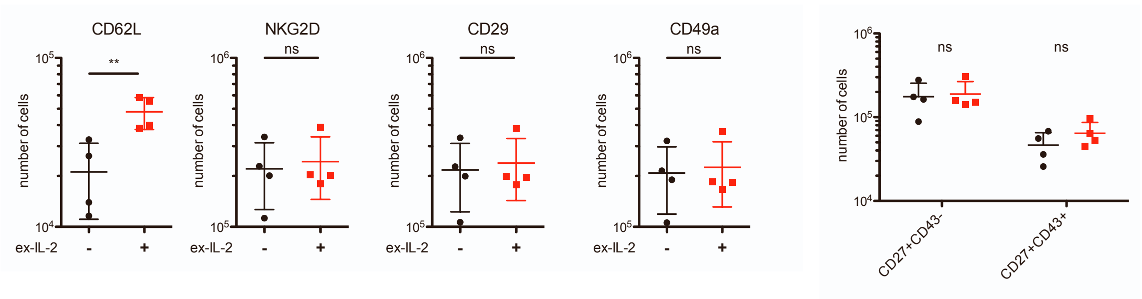


C

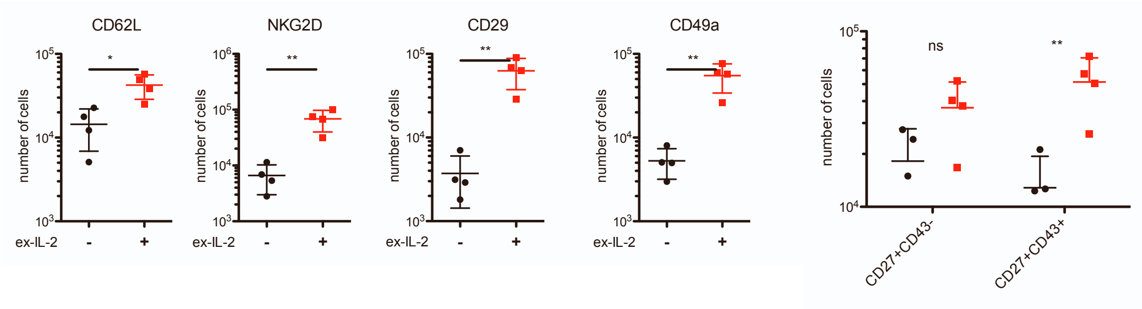


**Figure S3. ex-IL-2 promotes direct *in vivo* memory differentiation of *in vitro* activated cells transferred into naive mice, but has no impact on its potential to participate in an ongoing immune response. Related to Figures 3 and 4.**

CTV-labelled purified naive F5 CD8 T cells, at a concentration of $6x10^5$/ml ($1.5x10^5$/well), were cultured with CpG-matured, peptide-loaded cDC at a ratio of cDC:CD8 = 1:10 for 4 days. Divided CD8 cells were sorted by flow cytometry and $1x10^6$ cells were adoptively transferred into vaccinia virus-infected (4 days post-infection) (B) or naive (C) C57BL/6J mice.

**A.** Gating strategy for $CD45.1^+$ transferred F5 CD8 T cells is shown.

**B-C.** The number of cells expressing CD62L, NKG2D, CD29 or CD49a, and the number of $CD27^{+}CD43^{-}$ vs. $CD27^{+}CD43^{+}$ cells were measured on F5 CD8 T cells from spleen on day 32 after transfer into vaccinia virus-infected (B) or naive (C) mice.

One representative out of four independent experiments is presented. The results are expressed as the mean ± SD (n=4 mice per group). The statistical significance of differences was determined by the Student t-test (ns= $p>0.05$, *= $p\leq0.05$, **= $p\leq0.01$).

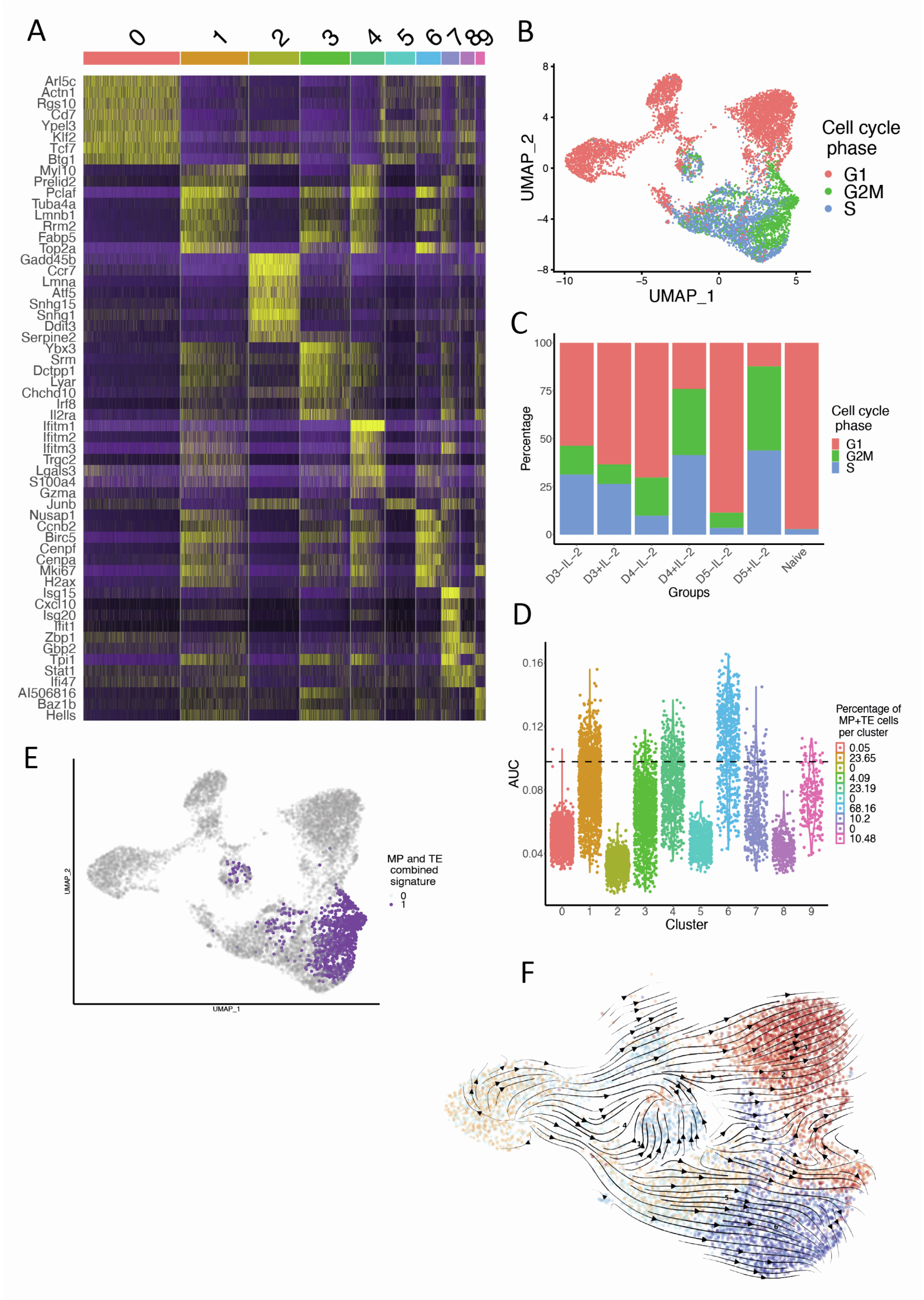


**Figure S4. Ex-IL-2 keeps CD8[+] T cells in a cycling state whereas they rapidly become quiescent in its absence. Related to Figure 5.**

**A.** Heatmap representing the top 8 differentially expressed marker genes for each cluster defined in Figure 5A.

**B-C**. Cells were classified into one of the cell-cycle phases (G1, S or G2/M) using the Seurat package and are colored accordingly. **B.** Cell-cycle position projected onto the UMAP. **C**. Percentages of cells in each cell cycle phase for each experimental condition.

**D.** Enrichment per cluster of combined memory precursor (MP) and terminal effector (TE) signatures. The dotted line represents the threshold above which, cells are considered positive for the combined (memory precursors + T effector cells) signatures. The legend indicates the percentage of positive cells in each cluster. AUC: area under the curve.

**E.** The cells with a combined memory precursor and terminal effector (TE) signature are colored on the UMAP.

**F.** RNA velocities of all activated CD8 T cells are projected onto the UMAP excluding naive cells (day 0).

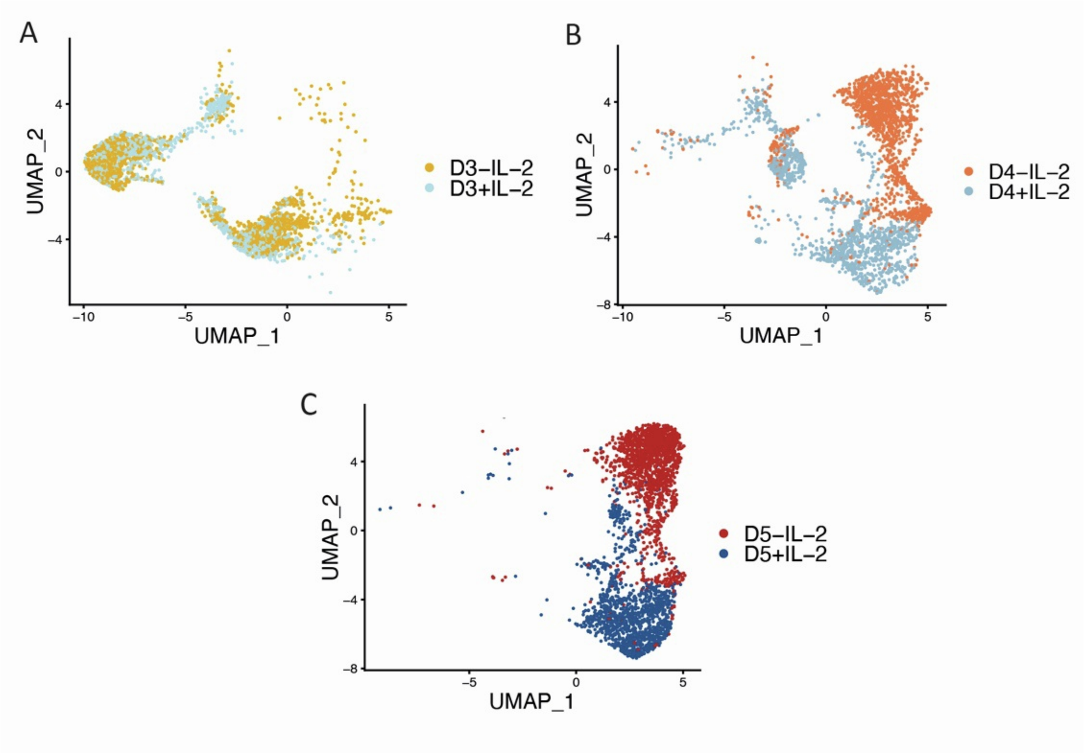


**Figure S5. The transcriptional programs of T cells activated with and without ex-IL-2 diverge after 4 days of activation. Related to Figure 5.**

UMAP projection of cells sorted on day 3 (A), day 4 (B) or day 5 (C) of activation with or without ex-IL-2. Cells are colored accordingly to the experimental time points and conditions of culture as in Figure 5B.

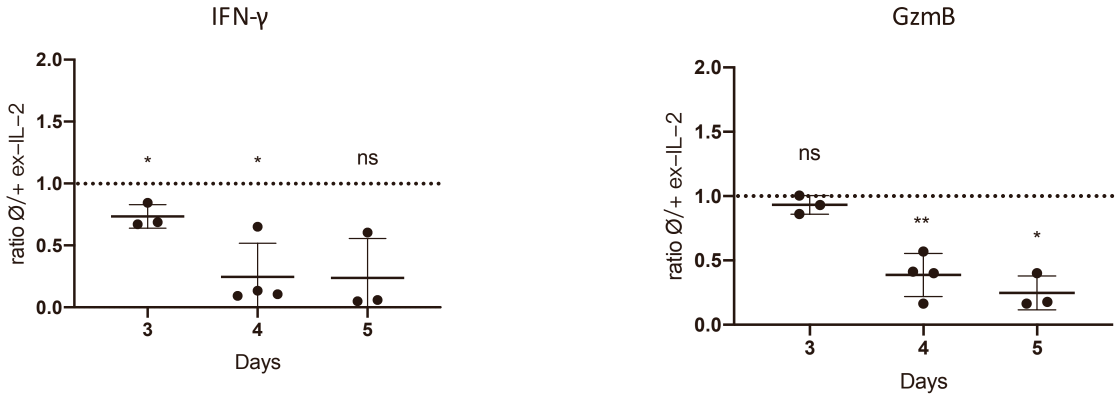


**Figure S6. The supplementation with ex-IL-2 sustains the expression of effector proteins by activated $CD8^+$ T cells. Related to Figure 6.**

$1.5x10^5$ magnetically purified naive F5 $CD8^+$ T cells labelled with CTV were activated with CpG-matured, NP68-loaded cDC at a ratio of cDC:CD8 = 1:10, in the presence or absence of ex-IL-2 (11,5 ng/ml) for 3, 4 or 5 days. After a 2h NP68 restimulation, percentages of IFN-$\gamma^+$ and $GzmB^+$ cells were measured and the ratio between cells cultured in the absence and cells cultured in the presence of ex-IL-2 was calculated. Values from three independent experiments for days 3-5, and four for day 4, are presented. The results are expressed as the mean ± SD. The statistical significance of the difference between the mean value of ratios and the hypothetical value of 1 was determined by the one sample t-test (ns= $p>0.05$, *= $p\leq0.05$, **= $p\leq0.01$).
